# Disturbed angiogenic activity of adipose-derived stromal cells obtained from patients with coronary artery disease and diabetes mellitus type 2

**DOI:** 10.1186/s12967-014-0337-4

**Published:** 2014-12-10

**Authors:** Nina A Dzhoyashvili, Anastasia Yu Efimenko, Tatiana N Kochegura, Natalia I Kalinina, Natalia V Koptelova, Olga Yu Sukhareva, Marina V Shestakova, Renat S Akchurin, Vsevolod A Tkachuk, Yelena V Parfyonova

**Affiliations:** Russian Cardiology Research and Production Complex, Moscow, Russian Federation; Faculty of Medicine, Lomonosov Moscow State University, Lomonosovsky av. 31/5, Moscow, 119192 Russian Federation; Institute of Diabetes Mellitus, Endocrinology Research Centre, Moscow, Russian Federation

**Keywords:** Adipose-derived stem/stromal cells, Cell therapy, Coronary artery disease, Diabetes mellitus type 2, Therapeutic angiogenesis, Plasminogen activator inhibitor-1

## Abstract

**Background:**

Multipotent mesenchymal stem/stromal cells (MSC) including adipose-derived stromal cells (ADSC) have been successfully applied for cardiovascular diseases treatment. Their regenerative potential is considered due to the multipotency, paracrine activity and immunologic privilege. However, therapeutic efficacy of autologous MSC for myocardial ischemia therapy is modest. We analyzed if ADSC properties are attenuated in patients with chronic diseases such as coronary artery disease (CAD) and diabetes mellitus type 2 (T2DM).

**Methods and results:**

ADSC were isolated from subcutaneous fat tissue of patients without established cardiovascular diseases and metabolic disorders (control group, n = 19), patients with CAD only (n = 32) and patients with CAD and T2DM (n = 28). ADSC phenotype (flow cytometry) was CD90^+^/CD73^+^/CD105^+^/CD45^−^/CD31^−^ and they were capable of adipogenic and osteogenic differentiation. ADSC morphology and immunophenotype were similar for all patients, but ADSC from patients with CAD and T2DM had higher proliferation activity and shorter telomeres compared to control patients.

ADSC conditioned media stimulated capillary-like tubes formation by endothelial cells (EA.hy926), but this effect significantly decreased for patients with CAD (p = 0.03) and with CAD + T2DM (p = 0.017) compared to the control group. Surprisingly we revealed significantly higher secretion of some pro-angiogenic factors (ELISA) by ADSC: vascular endothelial growth factor (VEGF) and hepatocyte growth factor (HGF) for patients with CAD and HGF and placental growth factor (PlGF) for patients with CAD + T2DM. Among angiogenesis inhibitors such as thrombospondin-1, endostatin and plasminogen activator inhibitor-1 (PAI-1) level of PAI-1 in ADSC conditioned media was significantly higher for patients with CAD and CAD + T2DM compared to the control group (p < 0.01). Inhibition of PAI-1 in ADSC conditioned media by neutralizing antibodies partially restored ADSC angiogenic activity (p = 0.017).

**Conclusions:**

ADSC angiogenic activity is significantly declined in patients with CAD and T2DM, which could restrict the effectiveness of autologous ADSC cell therapy in these cohorts of patients. This impairment might be due to the disturbance in coordinated network of pro- and anti-angiogenic growth factors secreted by ADSC. Changes in ADSC secretome differ between patients with CAD and T2DM and further investigation are necessary to reveal the MSC-involved mechanisms of cardiovascular and metabolic diseases and develop novel approaches to their correction using the methods of regenerative medicine.

**Electronic supplementary material:**

The online version of this article (doi:10.1186/s12967-014-0337-4) contains supplementary material, which is available to authorized users.

## Introduction

Cardiovascular diseases are the most frequent causes of death globally. The prevalence of metabolic disorders, first of all diabetes mellitus type 2, is similar to epidemic and greatly increases every year. Most of cardiac pathologies as well as T2DM result in permanent cardiac tissue damage followed by heart failure [1–4]. Current therapies primarily aim to treat the pathological remodeling of the heart that occurs after cardiac injury. However, there is a group of patients with refractory angina and heart failure that are incurable despite advances in medical treatment and surgical and percutaneous interventions. The attempt to find new treatment for these patients leads researchers to investigate the approaches of regenerative medicine using autologous and allogeneic stem cells.

Multipotent mesenchymal stromal cells (MSC) have been used in many animal and human studies and serve as an attractive candidate for tissue engineering and cell-based therapies. The neovascular potential of MSC is mostly supported by their ability to secrete potent pro-angiogenic growth factors and cytokines [5–8]. MSC can be derived from different tissue including bone marrow, adipose tissue, cord blood and umbilical cord, endometrium and some others. One of the most clinically relevant source of MSC is adipose tissue where these cells are located in perivascular niche [9–11]. There are numerous experimental studies and clinical trials which demonstrated the perspectives of using the adipose-derived stromal cells (ADSC) for the cure of different diseases [12–18]. ADSC are easily harvested, readily available and abundant in high numbers for the transplantation. In addition, soluble growth factors and cytokines secreted by ADSC may directly initiate vessels growth, branching and maturation, and also induce the release of angiogenic factors from other cell types [17,19–22]. In the perspective of cell-based therapeutic angiogenesis, there have been several clinical trials (ADVANCE, PRECISE, MyStromalCell Trial) that have showed some beneficial effects of ADSC therapy in patients with cardiovascular diseases [7,8,23,24]. However, overall therapeutic efficacy of the autologous MSC based cell therapy for the treatment of cardiovascular diseases is modest. It could be caused by the attenuation of MSC angiogenic potential in patients due to different risk factors affecting the stem and progenitor cell compartment. Some chronic diseases by itself also could attenuate MSC regenerative properties. In the present study we analyzed how ADSC properties are changed in patients with CAD and T2DM.

## Materials and methods

### Ethics statement

All procedures performed with tissue samples from patients were approved by Ethic Committee of the Russian Cardiology Research and Production Center. Each individual participate in the study signed a written informed consent for harvesting and using adipose tissue samples as well as for handling clinical data for research purposes.

### Patients

Nineteen patients without established cardiovascular diseases were included in the control group. They were underwent surgery because of general surgical pathology or endoprosthesis replacement of femoral or knee joints. Sixty patients who underwent coronary artery bypass grafting were divided into two groups of people with CAD only and with CAD + T2DM.

Exclusion criteria included autoimmune pathologies, cancer (even in the past history), acute or chronic inflammatory disease, acute myocardial infarction in the previous six months, long-termed hormone or antibiotics therapy, anemia (Hb < 10 g/dl) and hematological disorders, stroke or craniocerebral injury in the previous 12 months. Additional special exclusion criteria for the control group were any evidence for cardiovascular diseases and diabetes (based on standard clinical examination), including myocardial infarction or myocarditis, clinical signs of angina or heart failure, arrhythmias such as paroxysmal or permanent form of atrial fibrillation, frequent premature ventricular complexes, paroxysmal ventricular tachycardia, left bundle branch block, clinical manifestation of systemic atherosclerosis, severe dyslipidemia. Patients of both sexes were included in the study, but all women were under the post-menopause period and none of them received hormonal replacement therapy.

### Clinical data acquisition and analysis

Hospital in-patient databases were used to obtain clinical data including medical history, demographic characteristics (gender, age), diagnostic results, laboratory blood tests and medical treatment (Table 1). The body mass index (BMI) was calculated as the individual’s body mass divided by the square of his or her height (unit of measure of kg/m^2^). Data were interpreted as follows: normal if < 25, overweight if ≥ 25 and < 30, and obesity if ≥ 30. Severity of angina pectoris was estimated according to the criteria of the Canadian Cardiovascular Society Classification (CCSC) based on the extent of limitation on daily activities and the kind of physical activity which interrupted by the angina episode. Functional assessment of heart failure symptoms was made according to New York Heart Association (NYHA) classification. This system relates symptoms to everyday activities and the patient’s quality of life. All patients with CAD underwent left heart catheterization, left ventriculography and coronary angiography according to the guidelines for coronary angiography of the American College of Cardiology and the American Heart Association. Cardiac function was evaluated by global ejection fraction (EF). Global EF was estimated using respective software for different cardiac ultrasound systems: Vivid 7 (GE Ultrasound, Horten, Norway) and Philips-iE33 (Philips Medical Systems, Andover MA, USA). The extent of coronary artery atherosclerosis was scored as 0 (stenosis < 50%), 1 (stenosis of any main coronary artery > 50%), 2 (stenosis of two main coronary arteries >50%), and 3 (stenosis of three main coronary arteries > 50%).

**Table 1:** **Clinical characteristics of patients with coronary artery disease and diabetes mellitus type 2**

**Clinical characteristics**	**Groups of patients**			***p***
	**Control**	**CAD**	**CAD + DM2**	
Number of patients	19	32	28	-
Age, years	56,7 ± 12,3	62,3 ± 8,6	61,0 ± 8,2	NS
Gender, male, n (%)	5 (26,3%)	29 (90,6%)	17 (60,7%)	<0,05
Functional class of stable angina, n (%)	-	II FC-8 (25%),	II FC-5 (18%),	NS
		III FC - 13 (41%),	III FC-9 (32%),	
		IV FC – 11 (34%)	IV FC-14 (50%)	
MI in past history, n (%)	-	20 (63%)	18 (64%)	NS
The score of coronary artery atherosclerosis, n (%)	-	2CA-6 (18,8%)	2CA-5 (17,9%)	NS
		3CA-26 (81,3%)	3CA-23 (82,1%)	
Ejection fraction (%)	57,8 ± 1,8	55,0 ± 9,8	58,0 ± 9,2	NS
Arterial hypertension, n (%)	3 (15,8%)	27 (84%)	24 (86%)	<0,05
Obesity, n (%)	12 (63%)	20 (63%)	19 (68%)	NS
BMI, kg/m^2^	28,6 ± 3,7	30,6 ± 0,8	28,14 ± 1,07	NS
Total protein, g/l	68,7 ± 1,9	69,4 ± 1,6	72,1 ± 3,2	NS
Glucose, mmol/l	5,46 ± 2,2	5,4 ± 3,2	10,1 ± 4,1	<0,05
Triglyceride, mmol/l	1,7 ± 1,3	2,1 ± 1,1	1,9 ± 0,9	NS
Cholesterol, mmol/l	4,4 ± 0,4	4,5 ± 0,4	4,9 ± 0,9	NS
Urea, mmol/l	5,9 ± 0,8	6,2 ± 0,5	9,02 ± 1,5	NS
Creatinine, ummol/l	68,8 ± 9,5	66,6 ± 7,5	139,8 ± 23,2	<0,01
**Medical treatment before CABG**				
β-blockers, n (%)	-	27 (84%)	23 (82%)	NS
Antiplatelet agents, n (%)	-	32 (100%)	28 (100%)	NS
Statins, n (%)	-	28 (88%)	25 (89%)	NS
ACE inhibitors, n (%)	-	20 (63%)	18 (64%)	NS
Calcium antagonists, n (%)	-	13 (41%)	12 (43%)	NS
Diuretics, n (%)	-	23 (72%)	21 (75%)	NS
Nitrates, n (%)	-	27 (84%)	23 (82%)	NS

FC – Functional class, BMI – Body mass index, CABG – Coronary artery bypass grafting, p – p-value for multiple comparison of patient’s groups, NS – Non-significant.

### Isolation of adipose-derived stromal cells

Subcutaneous adipose tissue samples (0,5-5 ml) harvested during surgery were homogenized and digested in collagenase I (200 U/ml, Worthington Biochemical) and dispase (40 U/ml, Sigma) solution under agitation for 30–40 min at 37°C. Then tissue was centrifuged at 200 g for 10 min and the supernatant discarded. The pellet containing ADSC was lysed to destroy erythrocytes, filtered through a sieve (BD Falcon Cell Strainer, 100 mkm) and centrifuged at 200 g for 10 min. The final pellet was resuspended in culture medium. The cells were cultured in standard conditions (5% СО2; 37°С) in Advance Stem Cell Basal Medium (ADSCBM, HyClone) with 10% of Advance Stem Cell Growth Supplement (HyClone), 100 U/ml penicillin/streptomycin and 100 U/ml fungisone (HyClone). In 24 hours after isolation non-attached cells were washed off, and then medium was changed every 3–4 days. Cells yield was 4-7×10^4^ of attached cells/ml of tissue.

### Collection of ADSC conditioned medium

ADSC were harvested, using HQtase (HyClone), counted, washed by PBS and centrifuged at 4°C. Then ADSC pellet samples for RNA extraction were frozen in liquid nitrogen and stored at −70°С.

ADSC at 2nd passage were cultured to 70%– 80% confluence in culture dishes, then washed extensively with PBS, and replenished with supplement-free Advance Stem Cell Basal Medium for 48 hours. Collected media samples were centrifuged at 3,000 rpm for 10 min at 4°C to remove cell debris, and filtered through a 0.2 mm filter. Then conditioned medium was collected, supplemented with Protease Inhibitor Cocktail (1:500, Sigma) and frozen in aliquots at −70°С.

### ADSC surface markers expression analysis by flow cytometry

Cells were harvested with 2 mM EDTA/PBS and stained with antibodies against CD34 (BD Pharmingen), CD45 (BD Pharmingen), CD73 (BD Pharmingen), CD90 (BD Pharmingen), CD105 (BD Pharmingen), neuro-glial proteoglycan 2 (NG2, Chemicon), platelet-derived growth factor receptor B (PDGFRB, BD Pharmingen) conjugated with different fluorochromes, in appropriate conditions. Matching isotype control antibodies were used as negative control. The cells were analyzed using cell sorter MoFlo (Dako Cytomation).

### Differentiation

For osteogenic differentiation 60000 ADSC were seeded in collagen-coated 24-well plates with medium containing 10^−8^ M dexamethasone, 10 mM β-glycerol-2-phosphate and 5 μg/ml ADSCorbic-2-phosphate (Invitrogen) for 21 days. Mineralization of ECM was evaluated by staining with Alizarin Red S (40 mM, pH 4,2) (Sigma).

For adipogenic differentiation 60000 ADSC were seeded in 24-well plates with induction medium containing 10^−6^ M dexamethasone, 10 μM insuline, 200 μM indomethacine, 0,5 mM 3-isobutil-1-methylxantine (Invitrogen) for 6 days, then medium was changed to insulin-containing medium (10 μM) for 3 days followed by culturing in induction medium for 6 days. After 14 days of stimulation adipogenic differentiation was confirmed by Oil Red O staining for visualization of lipid droplets (Sigma).

ADSC from the same patient cultured in standard conditions without stimulation served as negative control.

### Proliferation

Proliferation of ADSC was evaluated using labeling with vital dye CFSE (Molecular Probes) according to manufacturer instructions. Its amount was diminished twice with every cell division. After 5 days labeled cells were analyzed by cell sorter MoFlo (Dako Cytomation) and their proliferation activity was determined using a specific formula as x_1_*1 + x_2_*2 + ….x_n_*n where x_n_ – percentage of cells in total population divided n times during 5 days.

At passage 1–2 the expansion of ADSC was also analyzed by using the trypan blue exclusion method. The number of PD (population doubling) was calculated by dividing the logarithm of the fold increase yield obtained at the end of the passage by the logarithm of 2.

### Relative telomere length measurement by quantitative real-time PCR (qRT-PCR)

DNA was isolated using Qiagen DNeasy Mini Kit (Qiagen) and concentration was measured at NanoDrop200 (Peqlap). Telomere length was measured by qRT-PCR and normalized to telomere length in HeLa cells [25].

### Quantitative telomerase activity assay

Cell pellets were resuspended in lysis buffer and analyzed with qRT-PCR using the quantitative telomerase detection kit (QTD, US Biomax) according to manufacturer instructions. Protein concentration was determined with the BCA Protein assay (Pierce). Telomerase activity of ADSC was expressed relative to HeLa cells and normalized to protein concentration for every sample. “Heat control” samples incubated for 15 minutes at 85°C served as negative control.

### Relative gene expression measured by qRT-PCR

Total RNA was isolated using RNase Mini Kit (Qiagen) followed by a reverse transcription reaction with Fermentas Reverse Transcription Reagents (Fermentas). RT-PCR was performed using SYBR Green PCR Master Mix (EuroGene) using BIO-RAD iQ5 Multicolor Real-time PCR detection system (Bio-Rad). PCRs in duplicates for every sample were performed in a final volume of 25 μl reaction mix that contained 50 ng of cDNA product, 100 nmol/L of each primer. For all reactions thermal cycling parameters were: 10 minutes at 95°C followed by 40 cycles of 15 seconds at 95°C, 20 seconds at 59-61°C depended on using primer and 20 seconds at 72°C. The oligonucleotides used as primers (EuroGene) are listed in Additional file 1: Table S1. GAPDH and β-actin were used as reference genes.

### Analysis of angiogenic factors accumulation in ADSC conditioned medium by ELISA

The conditioned media were analyzed for accumulation of vascular endothelial growth factor (VEGF), placental growth factor (PlGF), hepatocyte growth factor (HGF), angiopoetin-1 (ANG-1), angiogenin, thrombospondin-1 (THBS1) and plasminogen activator inhibitor-1 (PAI-1) using ELISA (R&D Systems) according to manufacturer instructions. Level of every protein was normalized to cell concentration for every sample of conditioned medium.

### In vitro tube formation assay

Effect of ADSC conditioned medium on capillary-like tubes formation on Matrigel in vitro was evaluated. Endothelial cells EA.hy926 [26] were seeded in 96-well plates coated with growth factor reduced Matrigel (BD Bioscience) in concentration 10^4^ cells per well and ADSC conditioned media were added. Three wells were used for each sample of conditioned medium. Supplement-free ADSC basal medium was utilized as a negative control; ADSC basal medium with 20% of Supplement served as positive control. For the blocking of PAI-1 effect the specific neutralizing antibodies (R&D Systems, 5 mkg/ml) were added to the corresponding wells. Plates were placed into CO2-incubator at 37°C and capillary-like structures were assayed in 24 hours under the light microscope (Leica, Germany). Total length of tubular structures was counted in 5 random fields of view per well (objective 10×) using MetaMorph 5.0 software (Universal Imaging). This parameter was used to characterize angiogenic activity of ADSC summary secreted products in vitro.

### Statistical analysis

Statistical analysis was performed using Statistica 8.0 software. Values are expressed as mean ± standard error of mean (SEM) for normally distributed data and median and percentiles (25%-75%) for not normal data. If normality of data was confirmed (according to Kolmogorov-Smirnov test and Shapiro-Wilk’s W test) comparison of independent groups was performed by Student t-test, if not or size of the analyzed sample was less than 10 cases – by Mann–Whitney U-criteria. Multiple comparisons were made using one-way ANOVA for normally distributed data, otherwise - by Kruskall-Wallis test. For dependent group comparisons the Wilcoxon rank sum test was used. For comparison of nominal variables distribution chi-square method was used. Correlation analysis was performed using Pearson correlation if both analyzed samples were normally distributed, otherwise Spearman correlation was applied. Statistical significance was defined as p-value <0.05 and all reported statistical tests were two-tailed.

## Results

### Clinical characteristics of the patients included in the study

In total, 79 patients were included in the study. The data of patients with cardiovascular diseases and T2DM are presented in Table 1. Nineteen patients from the control group didn’t have established cardiovascular diseases and metabolic disorders. The average age was 55,9 ± 2,3 (control group), 61,9 ± 8,9 (CAD), 61,3 ± 1,8 (CAD + T2DM) years and the differences between groups were not significant. There was a significant difference in the number of women between the control group (68,4%) and diseased patients (13% in CAD, 40% in CAD + T2DM). It is important to note that all women in the study were under the post-menopause period, and none of them received hormonal replacement therapy. However, to avoid the interference of gender differences between groups we compared the results obtained from men and women within every group of patients.

There were several obvious differences in characteristics of patients with CAD according to the presence or absence of T2DM. In particular, diabetic subjects had higher glucose and lower creatinine clearance in comparison to the patients with CAD only. Among the diabetic subjects 28% were on oral anti-diabetic agents only, 49% were on insulin with or without oral agents and 23% were on only anti-diabetic diet. Among the patients there was no difference in smoking, hypertension, BMI and the use of antiplatelet agents, statins, ACE inhibitors, beta-blockers, calcium channel blockers and nitrates.

### ADSC from patients with CAD and T2DM keep MSC characteristics

Culturing of cells harvested from adipose tissue as described above in the medium supported growth of undifferentiated mesenchymal stem cells allows to get at the 2^nd^-3^rd^ passages a relatively homogenous population of fibroblast-like cells with characteristics of MSC We assessed changes in cellular morphology of ADSC from patients with different pathologies through the first three passages. Under microscopic examination all the adherent cells displayed similar fibroblast-like spindle-shaped morphology. ADSC were examined by flow cytometry (Additional file 2: Figure S2, Additional file 3: Figure S3) and were found to be positive for CD73 (>85%), CD90 (>95%) and CD105 (>95%) with no or low expression of CD14 (<10%), CD19 (<10%), CD34 (<5%), CD45 (<1%), CD79 (<10%). ADSC also expressed pericyte markers like NG2 (>95%) and PDGFRB (>80%). Therefore, ADSC from healthy donors as well as from patients with CAD and T2DM express the minimal required immunophenotype criteria according to the International Society for Cellular Therapy Statement of minimal criteria for defining MSC [27].

One of the defining characteristics of MSC is an ability to differentiate into adiposytes, osteoblasts and chondroblasts. ADSC both from the patients without cardiovascular diseases and patients with CAD and/or T2DM demonstrated bone mineralization and neutral lipid accumulation in the appropriate culture medium and conditions, thus confirming the multipotent nature of ADSC obtained from investigated patients (data not shown).

**Figure 1:**
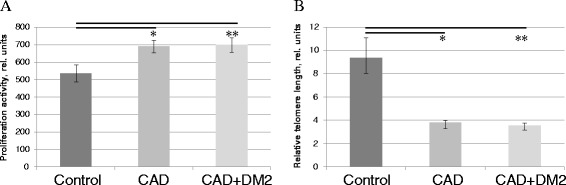
**ADSC from patients with chronic ischemic pathologies and metabolic disorders proliferate more actively and have shorter telomeres compared to the control group. (A)** Proliferation activity of ADSC evaluated by CFSE assay, data are mean ± SEM, *p = 0,048, **p = 0,046. **(B)** Relative telomere length in ADSC measured by RT-PCR, data are median and percentiles (25%-75%), *p < 0,0001, **p = 0,0004.

The effect of chronic pathologies on ADSC population doublings (PD) was calculated. We didn’t observe significant changes in the number of PD (passage 1–2) between ADSC obtained from patients with CAD (1,68 ± 0,08) and CAD + T2DM (1,53 ± 0,11) compared to the control group (1,47 ± 0,07), although there was a tendency to the increasing PD of ADSC from patients with CAD compared to the control patients (p = 0,11). In order to determine whether proliferation activity of different subpopulations of ADSC changed in the presence of CAD and T2DM we labeled cells with vital fluorescent dye CFSE and analyzed ADSC distribution according to CFSE intensity by flow cytometry. We couldn’t reveal any significant differences between studied groups of patients in ratio of active and slow proliferating cells subpopulations among the whole ADSC population, but we found that general proliferation activity of ADSC from patients with CAD and CAD + T2DM significantly increased compared to the control group (Figure 1A).

Proliferation activity of cells is closely related to the telomere length and telomerase activity. We evaluated relative telomere length and telomerase activity in ADSC from patients with and without CAD and T2DM. We observed significant decrease of the relative telomere length in ADSC from patients with CAD and T2DM compared to the patients from the control group (Figure 1B). We also measured telomerase activity in ADSC and detected it in all analyzed cell samples at the level about 5-fold lower than in HeLa cells. Measurement of telomerase activity in ADSC didn’t reveal significant changes between patients from different groups, probably because of high variability between samples. But there was no significant association detected between telomerase activity and relative telomere length in all groups of patients.

### ADSC from patients with CAD and T2DM have impaired ability to stimulate angiogenesis in vitro

**Figure 2:**
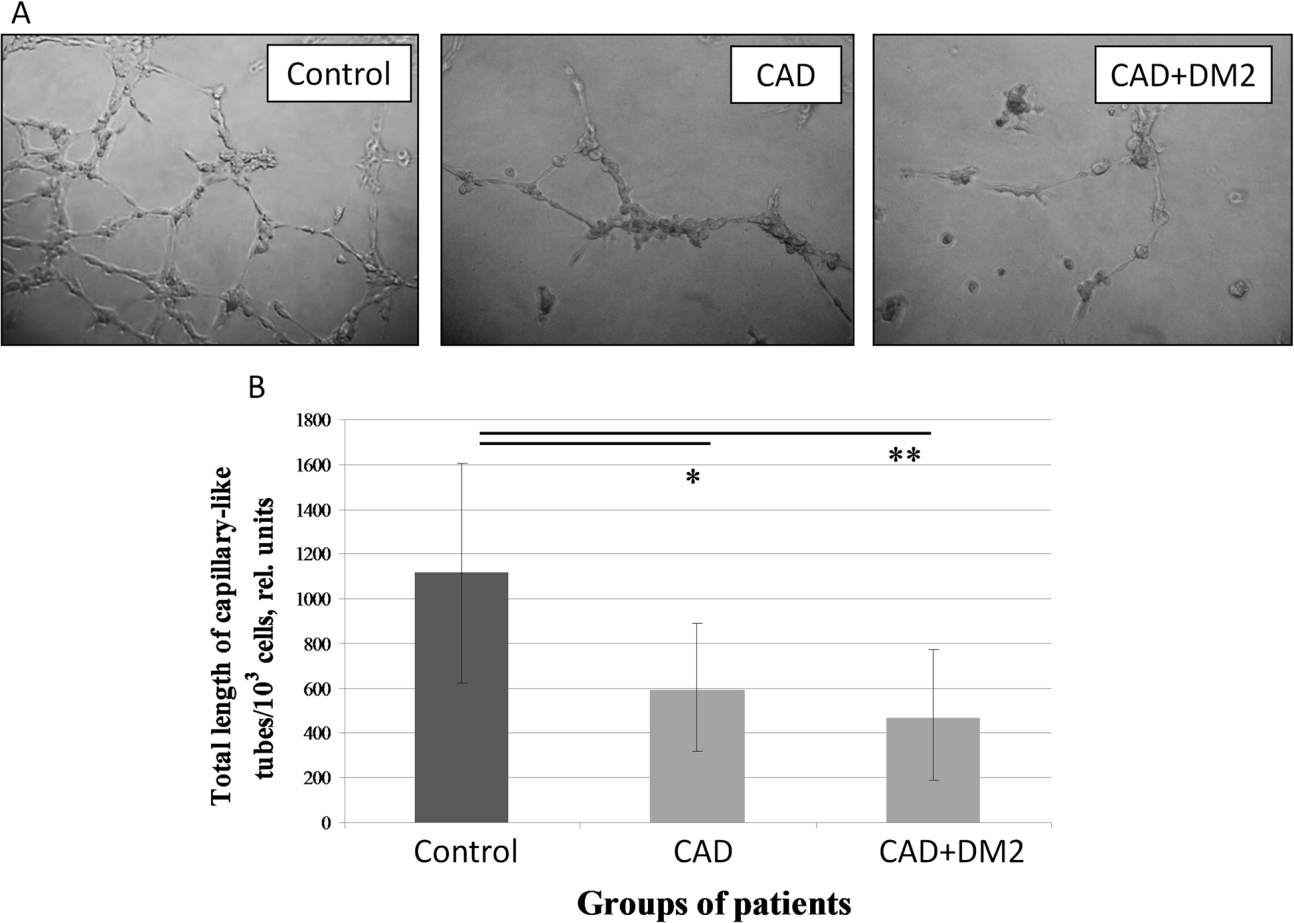
**Angiogenic activity in vitro of the summary products secreted by ADSC obtained from patients with CAD and DM2 decreased compared to the control group. (A)** Representative microphotographs of capillary-like tubes formed by EA.hy 926 endothelial cell line on Matrigel in the presence of ADSC conditioned media from the control group patient, patient with CAD and patient both with CAD and DM2. **(B)** Total tube length measurement for ADSC obtained from the patients with different pathologies, data are median and percentiles (25-75%), *p = 0,03, **p = 0,017.

Considering that the ability of ADSC to stimulate blood vessel growth is mainly mediated by paracrine mechanisms through the production of various angiogenic factors by these cells we analyzed angiogenic activity of summary products secreted by ADSC on the model of capillary-like tubes formation by endothelial cells on Matrigel. We found that ADSC conditioned media stimulated capillary-like tubes formation by endothelial cells (EA.hy926), but this effect significantly decreased for patients with CAD (p = 0.03) and with CAD + T2DM (p = 0.017) compared to the control group (Figure 2). There were no significant differences in ADSC angiogenic activity in vitro between from patients with CAD only and with CAD + T2DM (Figure 2). We also didn’t find any significant differences in angiogenic activity in vitro of ADSC obtained from men and women within every studied group.

**Figure 3:**
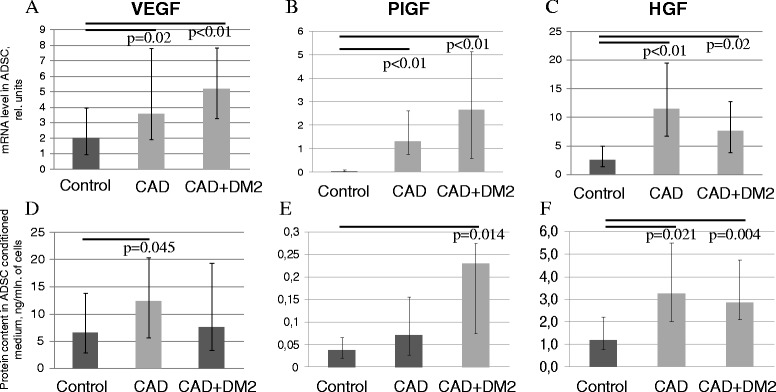
**Pro-angiogenic growth factor production by ADSC from patients with chronic ischemic pathologies and metabolic disorders was higher compared to the patients of the control group. (A-C)** Gene expression of VEGF **(A)**, PlGF **(B)** and HGF **(C)** in ADSC obtained from the patients with CAD, CAD + DM2 and the control group. Y-axis: mRNA level in ADSC, relative units. **(D-F)** Concentration of VEGF **(D)**, PlGF **(E)** and HGF **(F)** in ADSC conditioned medium. Y-axis: Protein content in ADSC conditioned medium, ng/mln. of cells. Data are median and percentiles (25-75%), p-values are presented on the graphs.

To reveal which angiogenic factors are involved in decreasing of angiogenic activity of ADSC summary secreted products in vitro in the presence of CAD and T2DM we analyzed gene expression of various angiogenesis-related factors in ADSC by real-time PCR. Surprisingly, we found that gene expression of such key proangiogenic factors as VEGF, PlFG and HGF was significantly increase in ADSC from patients with CAD and CAD + T2DM compared to the control group (Figure 3A,B,C). At the same time we didn’t observe significant disease-associated changes in gene expression of basic fibroblast growth factor (bFGF), angiopoetin-1, and angiogenin (data not shown).

Considering that level of secreted factors is more essential for paracrine effects of ADSC on angiogenesis we evaluated accumulation of these pro-angiogenic factors in the conditioned medium from ADSC. We found that ADSC obtained from patients with CAD secreted significantly higher amounts of VEGF and HGF and ADSC obtained from patients with CAD + T2DM secreted more PlGF and HGF compared to the control group which was consistent with the results of gene expression analysis (Table 2, Figure 3D,E,F). As for other angiogenic factors such as angiopoetin-1 and angiogenin we didn’t find significant changes in level of their secretion by ADSC dependent on the presence of CAD and T2DM (Table 2).

**Table 2:** **Accumulation of angiogenic factors in ADSC conditioned media (ng/mln. of cells) measured by ELISA**

**Growth factor**	**Groups of patients**			
	**Control**	**CAD**	**CAD + T2DM**	**p-value**
VEGF	6,55 (2,9-13,8)	**12,3 (5,6-20,3)**	7,52 (3,3-19,3)	*p = 0,045
HGF	1,19 (0,76-2,2)	**3,25 (2,0-5,5)**	**2,83 (2,12-4,75)**	*p = 0,021
				**p = 0,004
PlGF	0,04 (0,02-0,07)	0,07 (0,03-0,16)	**0,24(0,08-0,28)**	**p = 0,014
Angiogenin	1,62 (0,9-2,9)	1,8 (0,52-2,47)	0,74 (0,56-2,6)	NS
Angiopoetin-1	0,47 (0,15-0,9)	0,83 (0,28-1,7)	0,39 (0,18-0,67)	NS
PAI-1	40,06	**224,17**	**142,22**	*p = 0,0013
	(15,5-72,2)	**(110,2-322,8)**	**(45,0-268,5)**	**p = 0,0053
THBS-1	169,06	151,99	254,4	NS
	(59,7-585,1)	(57,3-382,1)	(85,1-421,8)	

*p-value for CAD group vs. the control group; **p-value for CAD + T2DM group vs. the control group; CAD – Coronary artery disease; T2DM- Diabetes mellitus type 2; NS- Non significant.

**Figure 4:**
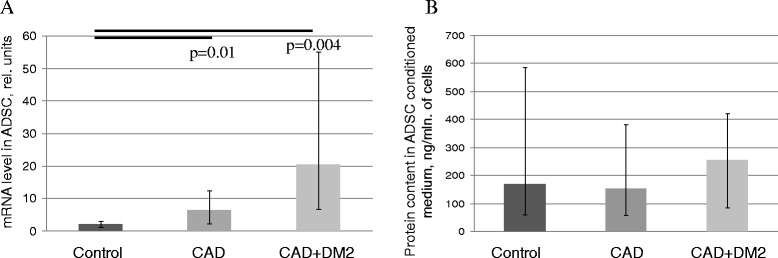
**Thrombospondin-1 (THBS-1) production by ADSC from patients with chronic ischemic pathologies and metabolic disorders compared to the control group. (A)** Gene expression of THBS-1 in ADSC obtained from the patients with CAD, CAD + DM2 and the control group. **(B)** Concentration of THBS-1 in ADSC conditioned medium. Data are median and percentiles (25-75%), p-values are presented on the graphs.

To explain the obvious discrepancy between the results of ADSC angiogenic activity in vitro assay and angiogenic factor production by these cells we considered that in the process of tissue regeneration, angiogenesis and homeostasis are supported due to the balance between pro-angiogenic and anti-angiogenic factors. So we analyzed the production of some angiogenesis inhibitors by ADSC from patients with different pathologies such as endostatin (ENDS) and thrombospondin-1 (THBS-1). We showed significant increase in mRNA level of THBS-1 in ADSC obtained from patients with CAD and with CAD + T2DM (Figure 4A). However, we failed to show the increase in the content of THBS-1 in the conditioned media of ADSC from the same patients (Table 2, Figure 3B). At the same time statistical analysis revealed negative correlation between mRNA level of TBHS1 in ADSC and their angiogenic activity measured by the tube formation assay in the group of patients with CAD (r = −0,59, p = 0,045) and in the group of patients with CAD + T2DM (r = −0,7, p = 0,008). As for ENDS we couldn’t find any significant differences both in mRNA level and protein secretion between ADSC obtained from patients with cardiovascular diseases and control subjects.

### PAI-1 suppresses in vitro angiogenic activity of ADSC from patients with CAD and T2DM

**Figure 5:**
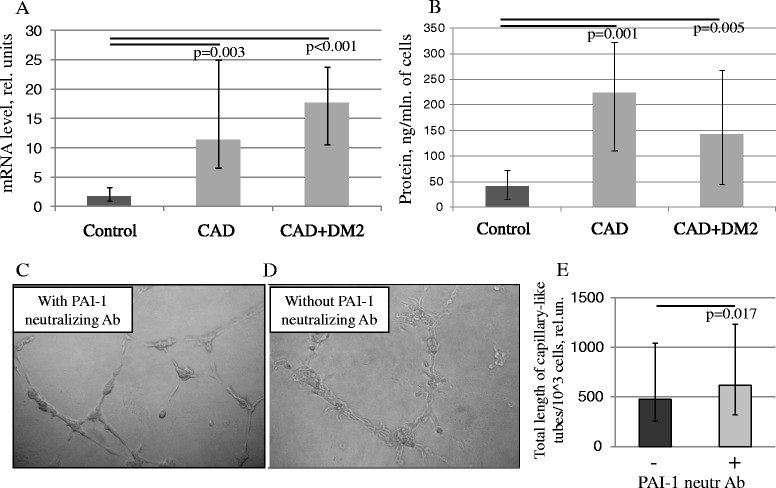
**Impact of PAI-1 to the decline of the angiogenic activity of ADSC obtained from the patients with CAD and CAD + DM2. (A-B)** PAI-1 production by ADSC from patients with CAD and DM2 increased compared to the control group. PAI-1 gene expression in ADSC **(A)** and protein concentration in ADSC conditioned medium **(B)** are presented. Data are median and percentiles (25-75%), p-values are presented on the graphs. **(C-D)** Representative microphotographs of capillary-like tubes formed by EA.hy 926 endothelial cell line on Matrigel in the presence of ADSC conditioned media (patient with CAD) with **(C)** or without **(D)** PAI-1 neutralizing antibodies (5 mkg/ml). **(E)** Total capillary-like tubes length measurement in the presence of PAI-1 neutralizing antibodies (5 ug/ml) in ADSC conditioned medium to the standard condition (without antibodies). Data are median and percentiles (25-75%), p-value is presented on the graph.

Apart from angiogenic growth factors effects extracellular matrix (ECM) remodeling is crucial for successful angiogenesis. ECM remodeling as well as directed cell migration for incorporation into forming vessel wall are regulated by some factors such as urokinase (uPA) and its receptor (uPAR), PAI-1 and matrix metalloproteases (MMP). Analyzing the gene expression of uPA, uPAR and PAI-1 in ADSC we found that mRNA level of PAI-1 was significantly increased in ADSC from patients with CAD and CAD + T2DM compared to control subjects (Figure 5A). These results were accompanied by data of ELISA which revealed higher concentration of PAI-1 in ADSC conditioned media obtained from the same patients in comparison to the control group (Table 2, Figure 5B).

To reveal the impact of PAI-1 to the angiogenic activity of ADSC summary secreted products in vitro we blocked PAI-1 effects by adding the specific neutralizing antibodies to ADSC conditioned medium samples and evaluating their ability to stimulate capillary-like tube formation (Figure 5C,D). We found that PAI-1 blocking in ADSC conditioned medium partially, but significantly restored the angiogenic activity of summary products secreted by ADSC from patients with cardiovascular pathology and diabetes (Figure 5E).

## Discussion

MSC, including ADSC, have been recently recognized as an attractive source of adult stem and progenitor cells for cell therapy and tissue engineering. Although the regenerative effects of these cells have been actively studied in various experimental models and clinical trials, little is known about the influence of chronic pathologies such as cardiovascular diseases and metabolic disorders on human ADSC. In the current study we analyzed ADSC obtained from patients with CAD and T2DM and assess how the disease itself could affect the properties of ADSC. It should be emphasized that we investigated namely ADSC as a promising tool for cell therapy, because these cells could be more easily and safer isolated from patients and considerably larger amounts of MSC could be obtained from adipose tissue compared to other sources of MSC such as bone marrow, etc. [28–30]. There is also some evidence that ADSC have a higher angiogenic and immunomodulatory capacities than bone marrow MSC and may be more suited for some clinical applications [30–32].

It should be noted that widespread risk factors associated with CAD (age, dislipidemia, obesity, smoking, arterial hypertension, glucose intolerance) may have an impact to the decrease of number and/or functional activity of stem and progenitor cells [33–36]. Previously we demonstrated that ADSC from aged patients with CAD keep MSC properties but exhibit age markers and have an impaired angiogenic potential [37]. Among the pathologies affecting the functionality of stem and progenitor cells some autoimmune diseases could be named such as systemic sclerosis and systemic lupus [38].

According to the results of several studies MSC number is unlikely decreased in patients with cardiovascular and metabolic diseases (such as T2DM), but their regenerative potential is essentially attenuated. Thus, Harris et al. [39] showed that number of ADSC obtained from 50 patients with different vascular pathologies was relatively consistent independently of age and comorbidities like obesity, T2DM, etc. [39]. Similar results were obtained by Madonna et al. [36] on the 42 patients with different grade of cardiovascular risk [36]. It was demonstrated that ADSC isolated from adipose tissue of patients with T2DM had lower proliferation activity and less responsive to the proangiogenic stimuli such as hypoxia [40]. In patients with obesity impaired differentiation potential of ADSC and decline of their ability to stimulate blood vessel growth were observed [41]. In the study performed by Vecellio et al. [42] MSC obtained from heart tissue of patients with T2DM were characterized by a reduced proliferation rate, diminished phosphorylation at histone H3 serine 10 (H3S10P), decreased differentiation potential, and premature cellular senescence compared to the control group [42].

In our study we showed that ADSC from the patients with CAD and CAD + DM had similar morphology and immunophenotype, preserved adipogenic and osteogenic differentiation potency, higher proliferation activity, but shorter telomeres than ADSC from the patients in the control group. Telomere shortening is often considered as a marker of replicative senescence of cells expanded in vitro, but in our study culture conditions and duration were similar for all samples, so we could assume that differences in relative telomere length were caused by patient-related factors. It might reflect the depletion of progenitor cell compartment in the presence of chronic pathologies such as CAD and T2DM.

Analyzing ADSC as a tool for therapeutic angiogenesis we found that angiogenic activity in vitro of ADSC obtained from patients with CAD and with CAD + T2DM decreased compared to the patients without established cardiovascular pathologies independently of such factors as age and gender. As paracrine mechanism is considered to be the main regulator of the beneficial effects of ADSC we examined the ability of ADSC to secrete some key angiogenic and antiapoptotic growth factors. Interestingly, we found that the level of VEGF, a critical vascular regulator, and important proangiogenic factor HGF secreted by ADSC were significantly higher for patients with CAD, as well as HGF and PlGF for patients with CAD + T2DM compared to the control group. Taking onto account that process of angiogenesis depends on the intricate balance between angiogenic and angiostatic factors, we speculated that the main cause of impaired in vitro angiogenic activity of ADSC obtained from patients with CAD and CAD + T2DM was the increased level of the angiogenesis inhibitors. We showed that gene expression of THBS-1, an inhibitor of angiogenesis through the direct effects on endothelial cell migration, proliferation, survival and apoptosis as well as by antagonizing the activity of VEGF [43], is significantly increased in ADSC from patients with CAD and CAD + T2DM. However, we couldn’t confirm this finding on the protein level pointing that some indirect mechanisms of THSP-1 participation in the angiogenic effects of ADSC could be discussed.

Analyzing the expression of factors involved in ECM remodeling and vascular cell migration and invasion we revealed that mRNA level of PAI-1 as well as its secretion by ADSC were significantly increased in groups of patients with CAD and CAD + T2DM. PAI-1 is one of the primary regulators of the fibrinolytic system, and it has a crucial effect on cell migration and adhesion. It was shown that high level of PAI-1 is linked with high risk of CAD, diabetes and obesity. PAI-1 can both promote and inhibit vascular remodeling, but its role in angiogenesis and tissue regeneration is still controversial. It may be presumed that the balance between these two mechanisms depends on a disease state. Plasma PAI-1 is closely correlated with such factors as hypoxia, glucose-related signaling molecules, inflammatory cytokines, triacylglycerol and insulin [44–46]. Acosta et al. [47] showed that ADSC obtained from patients with T2DM have less fibrinolytic ability as they secreted more PAI-1 and less tissue activator of plasminogen and D-dimer, which leads to the development of microtrombotic complications of cell therapy using these cells for the treatment of critical low limb ischemia [47]. PAI-1 actively interacts with uPA, an important factor of extracellular proteolysis, which not only specifically cleaves plasminogen and converts it into plasmin particularly causing different MMPs activation but also initiates intracellular signaling upon binding to its receptor on the cell surface and therefore plays a multiple role in vascular remodeling and angiogenesis. uPA system play an essential role in growth factor-induced endothelial cell migration and invasion. uPA gene transfer effectively induces functionally significant angiogenesis in models of acute MI and hind limb ischemia [48]. We suppose that PAI-1 produced by ADSC exerted anti-angiogenic effect also through the inhibition of uPA.

Importantly, we demonstrated that by neutralizing only one factor in ADSC conditioned medium – PAI-1 – one could partially restore the angiogenic activity of ADSC obtained from patients with chronic diseases. Our data are corroborated by the results obtained by Tashiro et al. [49] who have showed that PAI-1 inhibition in vivo under ischemic conditions increases the pro-angiogenic factor activity such as VEGF-A and FGF-2 and leads to the angiogenesis stimulation and improvement of tissue perfusion restoration [49].

It should be noted that we didn’t see any statistically significant evidence that T2DM had an additional impact to the decline of ADSC angiogenic activity in vitro when combined with CAD. There are numerical studies demonstrating that T2DM and particularly hyperglycemia as a main pathogenic factor of diabetes seriously affect MSC properties. Thus, impaired neovascular potential of ADSC obtained from streptozotocin-induced and db/db diabetic mice was demonstrated [50] as well as lower efficiency of these cells in promoting wound healing [51]. T2DM attenuated the capacity of MSC to promote neovascularization in the ischemic hindlimb which was secondary to hyperinsulinemia-induced, Nox4-dependent oxidant stress in db/db MSCs [52]. According to our data culturing of human ADSC in hyperglycemic conditions affects the ability of these cells to stimulate capillary-tube formation by endothelial cells in vitro [53]. However, in patients with CAD + T2DM the contribution of each disease to the influence on ADSC properties was hard to determine. Another issue was that ADSC isolated from patients with hyperglycemia were cultured ex vivo in normoglycemic conditions which could reverse some hyperglycemia-related effects on ADSC and mask the influence of T2DM on ADSC properties.

Considering the disease-associated dysfunction of ADSC obtained from patients with cardiovascular and metabolic pathologies one could make a conclusion that using allogeneic MSC from young healthy donors would be more appropriate approach for cell therapy in these cohorts of patients. However, there are some serious restrictions of this approach related to the immunogenicity and lack of engraftment of transplanted allogeneic MSC [54]. In the stem cell-based clinical trials both autologous and allogeneic cells are using in similar proportions [55] and there is still no clear evidence which source is better for clinical application.

We also have to highlight the necessity of extended investigation of ADSC secretome with the special emphasis on the balance between pro- and anti-angiogenic factors to characterize the individual angiogenic potential of ADSC obtained from patients, in particular, with cardiovascular and metabolic pathologies. The results of such studies will help to reveal some key factors (like PAI-1) that should be controlled to access the potency of autologous ADSC to stimulate angiogenesis. Another important challenge is to find the most promising targets for the correction of ADSC angiogenic properties before transplantation. ADSC could be easily modified by gene constructions contained angiogenesis-related factors, thus, VEGF-modified ADSC had significantly stronger therapeutic effect on the model of hind limb ischemia [56]. According to the results of our study PAI-1 might be a potential target for modification to modulate angiogenic activity of ADSC obtained from patients with CAD and T2DM. Another approach includes a hypoxic preconditioning as we showed that short-term hypoxia promoted ADSC ability to stimulate angiogenesis by coordinated changes in pro- and anti-angiogenic factor secretion by these cells [57].

## Conclusions

Taken together we showed that ADSC angiogenic activity is significantly declined in patients with CAD and T2DM, which could restrict the effectiveness of autologous cell therapy with ADSC in these cohorts of patients. According to our data, this impairment might be due to the disturbance in coordinated network of pro- and anti-angiogenic growth factors secreted by ADSC. We observed several changes in ADSC secretome between patients with CAD and T2DM pointing the possible specific targets for modification of autologous ADSC obtained from patients with different chronic pathologies to stimulate their regenerative potential. However, further investigations are necessary to reveal the MSC-involved mechanisms of cardiovascular and metabolic diseases as well as to develop novel approaches to their correction using the methods of regenerative medicine.

## Additional files

Additional file 1: Table S1. Sequence of primers used for real-time PCR. Additional file 2: Figure S1. Representative flow cytometry charts of MSC surface markers expression on ADSC obtained from patients with CAD, CAD+T2DM and control group. Red lines – specific antibodies, blue lines – isotype antibodies. Additional file 3: Figure S2. Representative flow cytometry charts of pericyte surface markers expression on ADSC obtained from patients with CAD, CAD+T2DM and control group. Red lines – specific antibodies, blue lines – isotype antibodies.

## Abbreviations

ADSC: Adipose-derived stem/stromal cells
CAD: Coronary artery disease
ENDS: Endostatin
EPC: Endothelial progenitor cells
GAPDH: Glyceraldehyde 3-phosphate dehydrogenase
HGF: Hepatocyte growth factor
MI: Myocardial infarction
NG2: Neuroglial proteoglycan-2
NO: Nitric oxide
PAI-1: Plasminogen activator inhibitor-1
PDGFRβ: Platelet-derived growth factor β-receptor
PlGF: Placental growth factor
T2DM: Type 2 diabetes mellitus
THBS1: Thrombospondin-1
uPA: Urokinase
uPAR: Urokinase receptor
VEGF: Vascular endothelial growth factor
