# Supplementary material for: Disturbed angiogenic activity of adipose-derived stromal cells obtained from patients with coronary artery disease and diabetes mellitus type 2
Source: J Transl Med. 2014 Dec 10;12:337. doi: 10.1186/s12967-014-0337-4 (PMC4268805; doi:10.1186/s12967-014-0337-4)
Supplement: Additional file 1: Table S1. — Sequence of primers used for real-time PCR. [file 12967_2014_337_MOESM1_ESM.docx]

**Supplemental Table -** Sequence of primers used for real-time PCR.

| **Gene** | **PCR product, bp** | **Sequence 5'-3'** |
| --- | --- | --- |
| VEGFA fw | 164 | CAACATCACCATGCAGATTATGC |
| VEGFA rv |  | GCTTTCGTTTTTGCCCCTTTC |
| PlGF fw | 124 | TGCGGCGATGAGAATCTGC |
| PlGF rw |  | AGCGAACGTGCTGAGAGAAC |
| HGF fw | 67 | AGGGGCACTGTCAATACCATT |
| HGF rv |  | CGTGAGGATACTGAGAATCCCAA |
| ANGPT1 fw | 136 | CTCGCTGCCATTCTGACTCAC |
| ANGPT1 rv |  | GACAGTTGCCATCGTGTTCTG |
| bFGF fw | 89 | AAGCGGCTGTACTGCAAAAAC |
| bFGF rw |  | TGAGGGTCGCTCTTCTCCC |
| Ang fw | 113 | CTGGGCGTTTTGTTGTTGGTC |
| Ang rw |  | GGTTTGGCATCATAGTGCTGG |
| uPA fw | 121 | TCAAAAACCTGCTATGAGGGGA |
| uPA rw |  | GGGCATGGTACGTTTGCTG |
| uPAR fw | 172 | TATTCCCGAAGCCGTTACCTC |
| uPAR rw |  | GGTGGCGGTCATCCTTTGG |
| PAI-1 fw | 109 | CATCCCCCATCCTACGTGG |
| PAI-1 rw |  | CCCCATAGGGTGAGAAAACCA |
| ENDS fw | 103 | GGCTGGCCTACGTCTTTGG |
| ENDS rw |  | CGGATGTGGAACAGCAGTGAG |
| THBS1 fw | 212 | CCTGACCGTCCAAGGAAAGC |
| THBS1 rw |  | CCTTTGCGATGCGGAGTCT |
| ActB fw | 144 | CCTGGCACCCAGCACAAT |
| ActB rw |  | GGGCCGGACTCGTCATAC |
| GAPDH fw | 187 | TGGTCACCAGGGCTGCTTTTA |
| GAPDH rw |  | TCCTGGAAGATGGTGATGGGATTT |
