# Supplementary figures and images for: Disturbed angiogenic activity of adipose-derived stromal cells obtained from patients with coronary artery disease and diabetes mellitus type 2
Source: J Transl Med. 2014 Dec 10;12:337. doi: 10.1186/s12967-014-0337-4 (PMC4268805; doi:10.1186/s12967-014-0337-4)

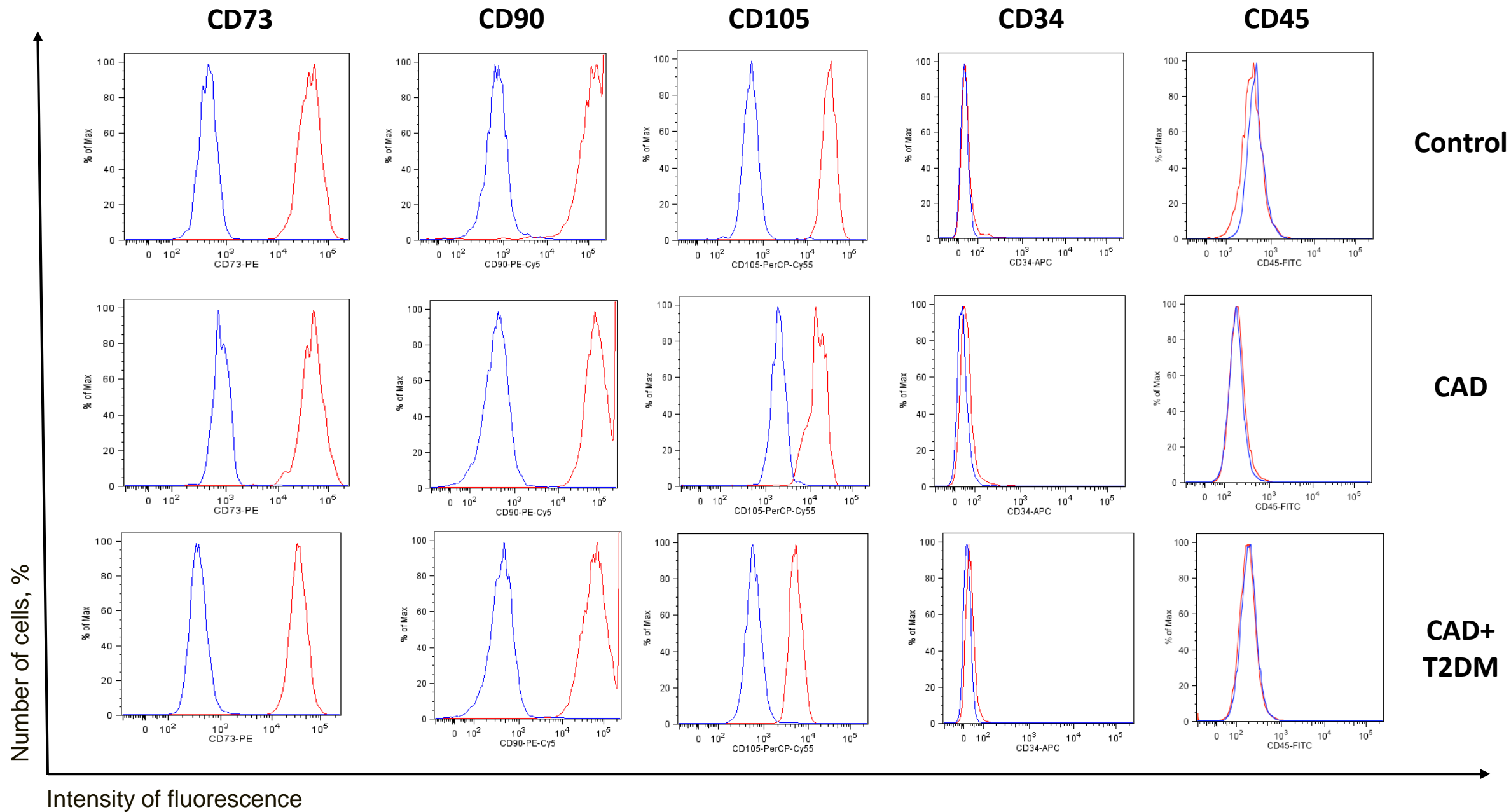

Supplement: Additional file 2: Figure S1. — Representative flow cytometry charts of MSC surface markers expression on ADSC obtained from patients with CAD, CAD+T2DM and control group. Red lines – specific antibodies, blue lines – isotype antibodies. [file 12967_2014_337_MOESM2_ESM.pdf]

**PDGFRB**

**NG2**

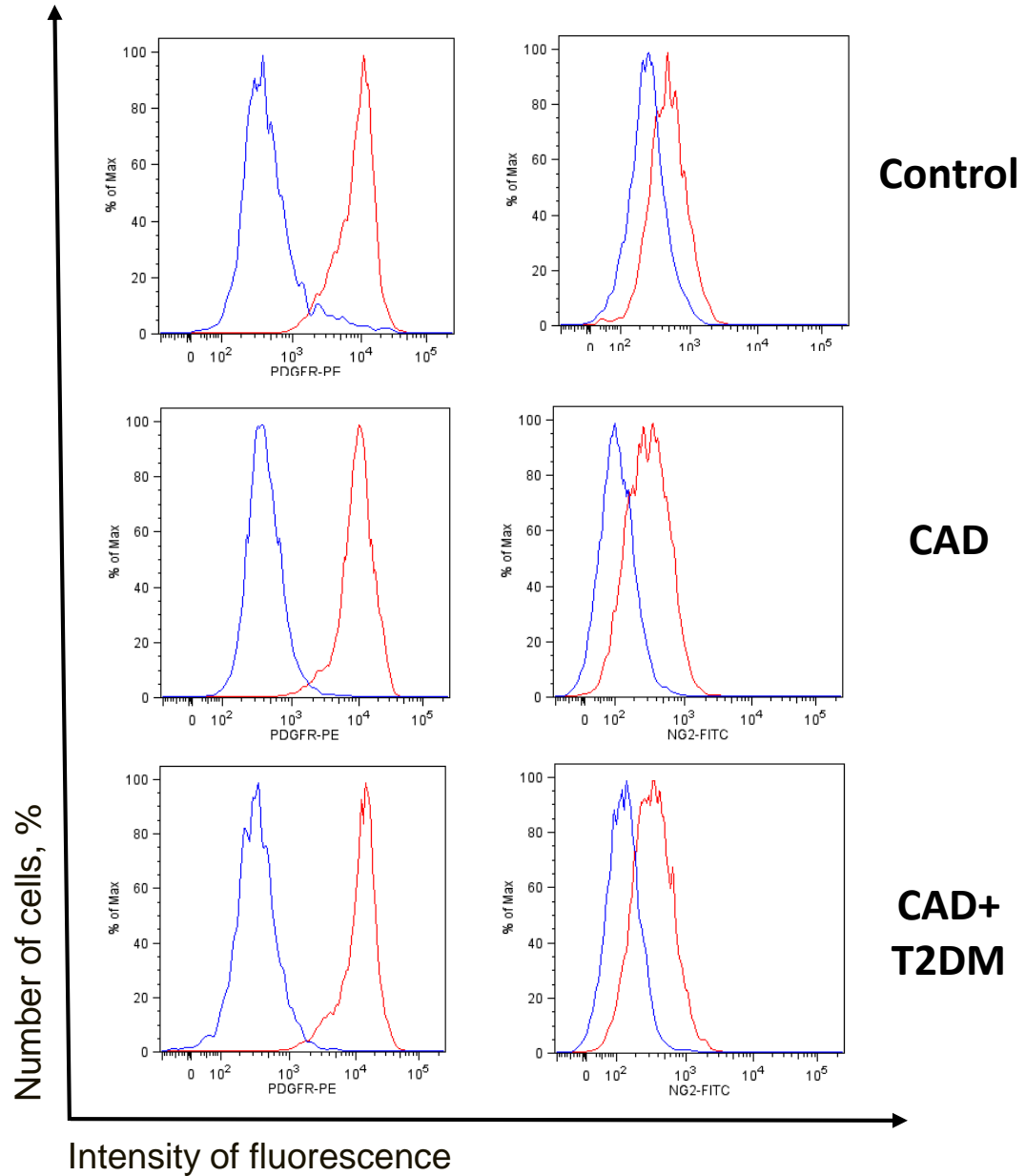

Supplement: Additional file 3: Figure S2. — Representative flow cytometry charts of pericyte surface markers expression on ADSC obtained from patients with CAD, CAD+T2DM and control group. Red lines – specific antibodies, blue lines – isotype antibodies. [file 12967_2014_337_MOESM3_ESM.pdf]
